# Dr. Corrigan’s Case of Urea Secreted in Large Quantity by the Peritoneum in a Case of Ascites

**Published:** 1842-08-06

**Authors:** 


*Urea secreted in large quantity by the Peritoneum in a case of Ascites.* By Dr. Corrigan.—A woman was attacked with dropsy of the abdominal cavity, for which she was tapped three times. The abdomen again enlarged, and with this she had many symptoms of Bright's disease of the kidney. The most remarkable feature of the case was, that the fluid which was drawn off contained urea in such large quantity, that Professor Kane, to whom the fluid was sent for analysis, could scarcely believe it was not urine.—*Dub. Journ. of Med. Sci.* March, 1842.

*On the Solubility of Fibrine and of Coagulated Albumen.* By M. Wohler.—M. Gmelin many years ago ascertained that coagulated albumen dissolved perfectly in water whose temperature was raised to 200° Cent. MM. Wohler and J. Vogel have made numerous experiments on the same subject, and ascertained that this solution takes place at 150°. They introduced coagulated albumen into tubes containing a certain quantity of water, closed them hermetically, and boiled them in oil at a temperature of 150° Cent.

The fibrine of the blood as well as muscular fibre boiled in water dissolved in the same manner; their solution was almost complete, little or no residue being left. Copious precipitates were thrown down from these solutions by means of acids; by nitric acid, even when it was very much diluted. The precipitate formed by acetic acid redissolved in an excess of the same.—*Edinburgh Med. and Surg. Journ.*, *from Journ. de Pharm.* March, 1842.

